# Dissociative Amnesia with Dissociative Fugue and Psychosis: a Case Report from a 25-Year-Old Ethiopian Woman

**DOI:** 10.1155/2020/3281487

**Published:** 2020-10-13

**Authors:** Liyew Agenagnew, Elias Tesfaye, Selamawit Alemayehu, Mathewos Masane, Tilahun Bete, Jinenus Tadessa

**Affiliations:** Department of Psychiatry, Faculty of Medical Sciences, Institute of Health, Jimma University, Jimma, Ethiopia P.O. Box 378

## Abstract

**Introduction:**

The case after exposure to intense traumatic events manifests signs and symptoms of dissociative amnesia with a dissociative fugue and schizophrenia. The psychotic symptoms we found, in this case, were very complicated and mimicking primary psychotic disorders. Therefore, this might be a good forum for the scientific world to learn from this case report, how psychotic disorders coexist with dissociative disorders, since the literatures in this area are too rare. *Main Symptoms and/or Important Clinical Findings*. This case report focuses on the case of dissociative amnesia with dissociative fugue and psychosis in a 25-year-old Ethiopian female who lost her husband and three children at the same time during the nearby ethnic conflict. Associated with amnesia, she lost entire autobiographical information, and she also had psychotic symptoms like delusions and auditory hallucination which is related to the traumatic event she faced. *The Main Diagnoses*, *Therapeutic Interventions*, *and Outcomes*. The diagnosis of dissociative amnesia with a dissociative fugue comorbid with schizophrenia was made, and both pharmacological and psychological interventions were given to the patient. After the intervention, the patient had a slight improvement regarding psychotic symptoms but her memory problem was not restored.

**Conclusions:**

The observation in this case report brings to the fore that individuals with dissociative amnesia with dissociative fugue can have psychotic symptoms, and it takes a longer time to recover from memory disturbances.

## 1. Introduction

Dissociation is a disruption, interruption, and/or discontinuity of the normal, subjective integration of potentially any aspect of experience and cognition, including behavior, memory, identity, consciousness, emotion, perception, body representation, and motor control which is primarily related to traumatic and/or overwhelming experiences (1). According to DSM-5, dissociative disorders are dissociative identity disorder, dissociative amnesia, depersonalization/derealization disorder, other specified dissociative disorders, and unspecified dissociative disorders (2). Dissociative amnesia with dissociative fugue is the “purposeful travel or bewildered wandering that is associated with amnesia for identity or for other important autobiographical information including awareness of time, awareness of self, and ability to mentally represent self-existence across time (3, 4). The memory impairment in dissociative amnesia is most frequently of a retrograde nature and is often limited to the episodic-autobiographical domain (4).

Dissociation is a rare disorder with prevalence estimated to be 0.2% (1, 4). The onset of this disorder is usually sudden and predicated by traumatic or stressful life events like physical trauma and/or psychologically stressful events, such as natural disasters (e.g., earthquakes and floods), marital discord, physical assault, personal threats, and war or military-related activities (5).

Patients with trauma-related disorders can have psychotic symptoms similar to those with a primary psychotic disorder (6, 7). So, this case report shows how a psychotic symptom presents in patients with the diagnosis of dissociative amnesia with a dissociative fugue.

## 2. Case Presentation

### 2.1. Patient Information

Mrs. S, a 25-year-old Ethiopian female, was brought to our hospital by her brother and her mother. She had no previous history of mental illness and hospitalization.

### 2.2. Case History

She was brought with a chief complaint of her inability to recognize her family and claiming that they were her enemies. On her presentation, she was very irritable, disturbed, and physically aggressive towards her brother and mother. She had poor self-care, unmade hair, and dressed in a disorganized manner. As her brother reported, she was found on the street while she was shouting and trying to beat others in the town three days before her presentation. When her brother got her, she claimed him as her enemy and threw a stone at him for which other people helped him to control her. Her brother said that he found her fourteen months after she left her home village. Fifteen months back, she faced a severe traumatic event following the ethnic conflict in her village, where she lost her husband and three children during that conflict.

Her brother reported that during the conflict she was in another nearby village, and when she came back to her home, she saw her husband and her three children (five-year-old son, three-year-old daughter, and 2-year-old son) slaughtered and all her properties destroyed. She also saw other people capturing a photo of the dead body of her husband and children's mutilated body parts. During that time, she shouted excessively but could not find any help even to bury them. She also phoned her elder sister living in another town and told her that her husband and children were murdered.

Since the conflict was on the spot, the police officers took everyone left in the village including Ms. S and her aunt to an internally displaced community's camp in another town and she did not take part in the funeral ceremony of her husband and children. At the refugee camp, she met with her elder sister whom she called on the phone. For the first three weeks in her stay at the internally displaced community's camp, she continuously cried, slept a few hours per day (less than usual), and spent more of her time alone. But after three weeks, she started to claim as if her husband and children have not died and as if they were somewhere else. She also began to refuse to take meals from her sister by claiming that she wanted to poison her. She became suspicious of her sister and her aunt and refused to sleep all night and tried to escape from the camp. Due to that she was restrained with metal chain and was given unknown traditional medication. She also started to claim that she was pregnant and she called the name of her first-born son and said as if he was in her uterus. She completely denied the death of her husband and her children and said that she never had a child. After she stayed in the camp for about six weeks, she escaped from there at night and could not be found in the town. Starting from the day she escaped from the camp, nobody knew her whereabouts and her family thought that she has died. Fourteen months later (3 days before her presentation to our clinic), her brother unexpectedly found her in another town which is about 1000 km far away from the internally displaced community's camp she escaped from. When he found her, she could not recognize him and claimed him as her enemy.

On her presentation, she often asked to have a cesarean section to give birth and she certainly believed that she was pregnant and could not give birth through spontaneous vaginal delivery because she believed as her enemies closed her uterus and the child became thin inside her uterus because she believed that her enemies suck her blood by using a mobile phone so that the child could not get enough blood from her. She said that she was wandering from town to town searching for a doctor who could help her to give birth. As she reported, she did not give birth to anyone, as if it was her first pregnancy. She claimed that she separated from her husband peacefully and reported as if he was somewhere else, and the name she recalled was not the real name of her deceased husband.

She did not remember where she was born, grew up, and overall information related to her background. And also, she did not remember how she traveled more than 1000 km away from her home village. Instead, she got a new name and did not respond when called by her previous name, place of birth, and family background. Otherwise, she had no family history of mental illness and medical history.

### 2.3. Physical Examination (PE) Findings

No abnormal finding was detected.

### 2.4. Results of Pathological Tests and Other Investigations

Urine HCG and abdominal ultrasound showed no pregnancy. Laboratory investigations like complete blood count, thyroid function test, liver function test, renal function test, lipid profile, and serum electrolytes were in the normal reference range. Head and neck CT scan was also done to rule out any biological cause, and it was normal. The Average Dissociative Experiences Scale (DES) was 66%.

## 3. Diagnosis

The diagnosis of dissociative amnesia with dissociative fugue comorbid with schizophrenia was considered based on the clinical findings and according to the DSM-5 diagnostic criteria of dissociative amnesia and schizophrenia.

## 4. Differential Diagnosis

Dissociative identity disorder, posttraumatic stress disorder, and major depressive disorder with psychotic feature were considered as a differential diagnosis for this case.

## 5. Therapeutic Intervention

### 5.1. Timeline and Patient's Progress in the Inpatient Unit

During the first week of her stay in the inpatient unit, she was disturbed, had difficulty falling and maintaining sleep, had frequent shouting by claiming that someone was capturing her photo in the absence of external stimuli, and often asked clinicians to have a cesarean section.

The first measure we took was calming the patient and ensuring her safety. For this reason, she was given risperidone 2 mg PO BID, diazepam 10 mg PO at night later. Risperidone was slowly titrated to 8 mg per day, and diazepam was titrated to 20 mg PO per day. The reason for titrating risperidone was to control psychotic disturbances of the patient, and diazepam was added to intervene with sleep difficulty.

In addition to this, occupational therapists consulted and evaluated the client. She took part in routine daily activities like making coffee and participating in conversation and plays. Even though the patient became calm sometimes, it was difficult to let her remember her autobiographical information. Any attempt to talk about the traumatic event she faced made her very irritable, and she often left the interview room. Sometimes, she became disturbed and cried by claiming that we refused her to help her in giving birth through cesarean section. She also claimed that she would kill herself unless we do so. Her medication (risperidone) was also changed to olanzapine due to the unavailability of risperidone, but the patient's psychotic experiences like delusional pregnancy, persecutory delusions, and somatic delusion were persistent and there was no improvement. Later on, after she took antipsychotics for six weeks, we discontinued it and put her only on fluoxetine and diazepam. Then, with a cooperative work with clinical psychologists, we let the patient talk with others and engage in social activities. At first, she was reluctant to actively engage in social activities. She was also given papers and colors to draw pictures and write what she wanted but she refused to do so even though she educated up to grade eight. Drug-assisted interview was also tried with phenobarbital 25 mg titrated up to 200 mg per day because amobarbital was not available. Additionally, the everyday schedule of hypnosis by different techniques was tried but it was difficult to let the patient into the suggestible state. However, the patient became somewhat interested especially during the hypnotic technique assisted by music and her interaction with clinicians and others improved. The patient's psychotic experiences and behavioral disturbances like shouting, crying, and frequent suicidal gestures persisted. For this reason, prescribing antipsychotics (olanzapine 10 mg titrated till 20 mg PO per day) combined with scheduled psychological interventions was reconsidered. Additionally, since the patient refused to take oral medication, long-acting Fluphenazine Decanoate 25 mg IM injection was given for her. Furthermore, we used persuasion and suggestive techniques and tried to give a sense of safety and security. We let the patient engage more in daily activities like washing her mother's and her clothes, making coffee, cleaning the room, and others. After that, she became behaviorally calm and cooperative for doing activities given for her, she started to give care for her 6-year-old nephew whom she previously claimed her enemy. She also started to help other patients and her relationship with her mother and her brother improved even though she did not believe that they were her families. She reported that they were no longer her enemy, and she could live with them smoothly. The patient was discharged after a five months stay in the hospital and appointed for close follow-up. The patient's overall autobiographical memories are not still fully retrieved, and we are following her for further interventions by keeping her in the community.

The summary of the patients' progress in the inpatient and outpatient unit is described below in Tables 1 and 2.

## 6. Discussion

This paper describes the patient who has suffered from generalized retrograde amnesia for her entire autobiographical information which supposedly resulted from a severe traumatic event she experienced due to ethnic conflict and loss of her husband and her three children during the conflict. The patient's memory impairment is the autobiographical memory system (8) and social abilities preservation even though the patient was reluctant to engage in social activities. She lost overall information related to her identity including her own and parents' names, birth date, and place of birth, and she had no information about the trauma that happened to her. She did not remember how she traveled more than 1000 km away from her home village and even she denied whether she traveled all this way and said she was born here.

The patient became very irritable while she was asked for any information about her previous identity and instead she had a new name with well-confabulated information about her new identity. This might be due to a severe and troublesome trauma that happened to her families which is awful to remember. The patient also had psychotic experiences like persecutory delusion, a delusion of pregnancy, and auditory and visual hallucination, and the patient reported that the voice she heard came from inside her and this could be conceptualized as dissociative rather than psychotic and supported by previous studies (7, 9). But her thought form was coherent and logically connected. She also had a depressed mood and cried occasionally. Physical and neurological examinations excluded other somatic problems associated with organic amnesia (10). Malingering is unlikely because of a lack of intelligible reward she could get from simulation for such a long period (11, 12). Posttraumatic stress disorder (PTSD) is one of the psychiatric disorder which could be a psychological reaction to such severe trauma (13) However, this patient had no disturbances like flashbacks, nightmares, intrusive memories of the trauma, and persistent avoidance of stimuli associated with the traumatic event; rather, she completely forgot everything related to that the traumatic event not only the important aspect of the traumatic event; because of this, we ruled out putting posttraumatic stress disorder as a primary diagnosis. Another important differential diagnosis was dissociative identity disorder because she has recurrent gaps in the recall of everyday events, important personal information, and/or traumatic events that are inconsistent with ordinary forgetting which is one of the diagnostic criteria but she did not fulfill other DID criteria's like having two or more distinct identities, so we did not consider as the main diagnosis. And also, she did not fulfill the DSM diagnostic criteria of major depressive disorder even if she had some clinical manifestations like sleep disturbance, recurrent suicidal ideation, and occasional crying associated with the delusion she had.

The hallmark dissociative symptoms in this patient were the inability to recall important autobiographical information associated with a traumatic event that is too extensive to be explained by ordinary forgetfulness, and it was generalized amnesia for identity and life history and sudden and unplanned travel away from the camp. Also, she had full-blown psychotic symptoms more than a year as explained in the case presentation section. Due to this, we made a diagnosis of dissociative amnesia with dissociative fugue comorbid with schizophrenia. However, in previous studies, there has been a controversy about whether the psychotic experiences might be due to conversion and defense against underlying troubling trauma or an independent psychotic phenomenon.

The retrograde amnesia of this patient was presumably triggered by a severe traumatic event from an ethnic conflict from which more than one thousand people died including her husband and her three children and about 1.5 million people displaced. Psychotherapeutic interventions are recommended for treatment of dissociative amnesia and positive outcomes were reported (14–16), and there was an improvement in behavioral disturbances like aggressiveness and suicidal behaviors for this particular patient following every day scheduled hypnosis and social engagement in social skill training. This patient had psychotic disturbances, and atypical antipsychotic drugs may be of use in treating complex trauma cases with psychotic symptoms (10), and for this particular case, risperidone and olanzapine were tried and because of her nonadherence to PO medications; she was put on modicate 25 mg IM monthly, and also, she got antidepressants and benzodiazepines to alleviate the emotional and dissociative symptoms. She got some improvement even though the psychotic symptoms are not completely remitted. Her delusion of pregnancy especially persisted, and the previous study suggests that delusion of pregnancy might take a longer period to remit (17).

The patient has experienced a different type of side effects including neuroleptics-induced pseudo-Parkinson related to the usage of those antipsychotic medications which might be because of using high dosage and in the availability of quetiapine in our set up which is the suggested antipsychotic medication for patients with dissociation in previous studies with low side effect and high efficacy (18). We understood that using antipsychotics is necessary to calm the patient and treat the psychotic symptoms but to avoid side effects of the medication by measuring the risk-benefit of using a low dosage of antipsychotic medication is preferable in addition to different psychological interventions modalities.

Patients with dissociative amnesia can regain their lost memory early, and for some of them, it might take several years to recover (5, 14, 19). In this case, at this time, it is difficult to predict whether she regains her identity and objective facts associated with her life history.

## 7. Conclusions

This patient has suffered from long-lasting retrograde amnesia of her life with impaired self-identification which is associated with severe psychotic disturbances. It is rare to get dissociative amnesia associated with psychotic symptoms, and this is an exceptional case.

Therefore, clinicians should be aware and give due attention to find such disturbances in the patient who presented with amnesia and severe traumatic experiences.

Lastly, it is important to investigate the possible relationship between medications, dissociative symptoms, and psychotic symptoms further in follow-up studies that could explain certain details.

## 8. Strength and Limitations of the Case Report

Contribution of multidisciplinary teams in this case report and admitting the patient to a psychiatric ward to follow each sign and symptoms were the strength of the case report, whereas scarcity of literatures in this issue and lack of standard guideline on how to manage patients with dissociation and psychosis were the major limitations that we identified in this case report.

## 9. Patient Perspective

She reported that she has improvement regarding her sleep, appetite, and calm behavior and is supportive of the treatment and ready to take medications cooperatively.

## Figures and Tables

**Table 1:** Summary of the patient's progress in the inpatient unit.

Duration	Psychopathology and main lab findings	Therapeutic interventions		Remark
		Pharmacological	Psychosocial	
First and the second week	-The patient was hostile, disturbed, and had frequent shouting She did not know where she was and where she was brought from-Lab investigations like CBC, TSH, RFT, and LFT were normal-HCG, negative-Dissociative experiences scale: 66	-Risperidone 2 mg PO BID -Diazepam 10 mg PO noct In the second week, fluoxetine 20 mg PO in the morning was added	Rapport building and empathic understanding Psychoeducation on drug adherence and side effects	Dx: dissociative amnesia with dissociative fugue comorbid with schizophrenia
Third week	-Abdominal U/S was done and confirmed, as she is not pregnant.-The patient becomes more engaging but very irritable when asked about the trauma-Dissociative experiences scale: 66	-Risperidone titrated to 6 mg/day-Diazepam was tapered and discontinued-Artane 2 mg PO morning	-Engaging the patient in scheduled routine daily activities	Slight improvement(behaviorally) but a present of neuroleptic-induced Parkinson
Fourth week	-Reported suicidal ideation and tried to strangulate herself-SAD PERSONAS = 9/16-Had wrist rigidity and drooling of saliva	-Risperidone titrated to 8 mg/day then to 10 mg/day-Fluoxetine titrated to 40 mg/day	-emphatic understanding and encouragement of social relationships with other patients and attendants	-Worsening-24 hours under close follow-up
Fifth and sixth week	-Very irritable-Frequent shouting-Hostile towards her mother-Drug side effects observed-Dissociative experiences scale: 66	-Risperidone tapered slowly until 4 mg/day diazepam 10 mg PO BID reinitiated-Fluoxetine tapered to 20 mg/day	Engagement in routine daily activities-hypnosis	-An additional diagnosis of neuroleptic-induced pseudo-Parkinson
		
Seventh week	-Simpson Angus scale = 16	-Antipsychotic discontinued-Diazepam 10 mg PO BID-Fluoxetine 20 mg/day	-Hypnosis-Engagement in routine daily activities like washing clothes, and making coffee	-Dx: dissociative amnesia with dissociative fugue, comorbid with schizophrenia-Neuroleptic-induced Pseudo parkinsonism
Eighth and ninth week	-More engaging	-Additionally, phenobarbitone was initiated for drug-assisted hypnosis and titrated to 100 mg PO BID	-Drug-assisted hypnosis-She was let to engage more in daily activities	Improving
Tenth week	-Head and neck CT was done and normal finding-Abdominal U/S confirmed no pregnancy-Hostile-Suicidal and homicidal towards her mother-crying-SAD PERSONAS = 7/16	The same	-Hypnosis continued every day-EMDR	Close follow-up for possible suicidality and homicide
Eleventh week	-Sleeplessness-Shouting-Physical and verbal aggression-Severe paranoia towards everybody	-Risperidone 2 mg PO BID reinitiated-Haloperidol 5 mg IM Prn-Phenobarbitone tapered and discontinued-Fluoxetine and diazepam continued	-Engagement in social activities	-Worsening-Close follow-up
Twelfth week	-Acute dystonia torticollis)	-Risperidone changed to olanzapine 5 mg PO BID due to unavailability	-hypnosis continued-Encouragement of engagement in routine daily activities	Slight improvement (behaviorally)
Thirteenth week	No new finding	-Fluoxetine was changed to amitriptyline due to unavailability	-Hypnosis continued-Encouragement of engagement in routine daily activities	
Fourteenth week and fifteenth	-refusal to take medication	-Modicate 12.5 IM testing dose-Amitriptyline was titrated to 100 mg PO noct-Olanzapine titrated to 20 mg PO/day	-Hypnosis continued-encouragement of engagement in routine daily activities	
Sixteenth week	-A frequent complaint of constipation	Bisacodyl 5 mg PO BID added	-Hypnosis continued-Encouragement of engagement in routine daily activities	Follow for possible drug side effect
Seventeenth week	-Constipation continued despite treatment	-Enema was done-Modicate 25 mg IM given	-Hypnosis continued-Encouragement of engagement in routine daily activities	
Eighteenth week	-Good social interaction	-Amitriptyline was changed to sertraline through cross tapering	-Hypnosis continued-Encouragement of engagement in routine daily activities	Improving
Nineteenth week	-Good social interaction-Good interaction with her mother but not accepted as her true mother	-Sertraline 100 mg PO morning-Diazepam 10 mg PO BID-Olanzapine 5 mg PO BID-Engage in routine daily activities	-Hypnosis continued-Encouragement of engagement in routine daily activities	Improving
Twentieth week	-Improved engagement in social activities like helping other patients-She said her mother is no more her enemy	-Diazepam tapered-Sertraline was discontinued-Discharge considered	-Encouragement of engagement in routine daily activities	-Improving

Follow-up and **o**utcomes.

**Table 2:** Patient's progress at outpatient.

Follow-up visits	Clinician-assessed outcomes	Patient-assessed outcomes	Important follow-up test results (positive or negative)	Intervention adherence and tolerability (and how this was assessed)	Adverse and unanticipated events
Two weeks after discharge	Calm, well dressed, cooperative	She had good sleep, willing to engage in routine daily activities, and smoothly living with her mother and nephew.	None	Adherent to medication (modicate and olanzapine)	None
Four weeks after discharge	Calm, no memory of her autobiographical information	Improved engagement in routine daily activities	None	Adherent to medication and no side effect reported or observed	None

## Data Availability

Data sharing does not apply to this article as no datasets were generated or analyzed during the current study. Ethical Approval Ethical clearance was obtained from the ethical review board of Jimma university institute of health.

## Abbreviations

CBC: Complete blood count
BID: Bis in die or twice daily
DES: Dissociative experiences
HCG: Human chorionic gonadotrophin
IM: Intramuscular
LFT: Liver function test
Noct: Nocturnal or night time
PTSD: Posttraumatic stress disorder
RFT: Renal function test
SAS: Simpson Angus Scale
TSH: Thyroid-stimulating hormone.
